# Mdivi-1 Attenuates Sepsis-Associated Acute Lung Injury by Inhibiting M1 Alveolar Macrophage Polarization and Pyroptosis

**DOI:** 10.1155/mi/3675276

**Published:** 2025-03-30

**Authors:** Xiaoyu Zhang, Hui Fan, Li Su, Yanni Wang, Guozhong Chen

**Affiliations:** Department of Respiratory and Critical Care Medicine, Renmin Hospital of Wuhan University, Wuhan, Hubei 430060, China

**Keywords:** acute lung injury, DRP1, M1 alveolar macrophage polarization, Mdivi-1, NLRP3 inflammasome, pyroptosis

## Abstract

**Background:** Dynamin-related protein 1 (DRP1)-dependent mitochondrial fission is a novel target for mitigating inflammatory diseases. This study aims to explore the effects of the DRP1 inhibitor Mdivi-1 on sepsis-induced acute lung injury (ALI).

**Methods:** C57BL/6 mice were intraperitoneally injected with lipopolysaccharide (LPS) and then treated with or without Mdivi-1 2 h post-injection. RAW264.7 alveolar macrophages were stimulated with LPS and treated with or without NLRP3 inhibitors, Mito-TEMPO, or Mdivi-1. Hematoxylin and eosin (H&E) staining was used to observe pathological changes in lung tissues. The levels of inflammatory cytokines in lung tissue homogenates, serum, and cell culture medium were detected using enzyme-linked immunosorbent assays (ELISA). The mRNA expression of macrophage polarization markers, NLRP3 activation, and phosphorylation status of DRP1 were assessed. Flow cytometry was employed to evaluate the levels of macrophage apoptosis. Immunofluorescence was utilized to detect the levels of in vivo and in vitro macrophage polarization markers. Mitochondrial reactive oxygen species (Mito-ROS) were measured using a Mito-SOX assay kit.

**Results:** Our results suggested that Mdivi-1 reduced lung tissue pathological injury, M1 alveolar macrophage polarization, NLRP3 activation, and DRP1 Ser616 phosphorylation. In vitro, LPS triggered abnormal accumulation of M1 polarization, NLRP3 activation, and excessive increase in Mito-ROS. NLRP3 inhibitors and Mito-TEMPO inhibited M1 alveolar macrophage polarization and pyroptosis-mediated tissue damage. Mito-TEMPO significantly inhibited NLRP3 activation. Furthermore, Mdivi-1 reduced ALI by inhibiting M1 polarization and pyroptosis. The mechanism of Mdivi-1 in reducing M1 alveolar macrophage polarization and pyroptosis may be related to the inhibition of DRP1-mediated mitochondrial fission, thus suppressing the Mito-ROS/NLRP3 pathway. Similar results were observed *in vitro* by knocking down DRP1.

**Conclusion:** Inhibition of DRP1 by Mdivi-1 alleviates ALI by hindering Mito-ROS/NLRP3-mediated M1 alveolar macrophage polarization and pyroptosis, suggesting that DRP1-dependent mitochondrial fission is a potential therapeutic target for ALI.

## 1. Introduction

Acute lung injury (ALI) is an acute, diffuse, inflammatory pulmonary insult, characterized by increased alveolar-capillary permeability, augmented lung mass, and diminished pulmonary ventilation, commonly leading to multi-organ failure and mortality [1, 2]. ALI is marked by acute respiratory dysfunction accompanied by rapid, refractory hypoxemia, reduced lung compliance, and diffuse alveolar damage as evidenced by chest X-ray [3]. These alterations promote the activation of immune cells within the body, leading to aberrant immune responses such as the accumulation of immune cells and the release of proinflammatory mediators, chemokines, and proteases, ultimately progressing to acute respiratory distress syndrome [4]. Despite significant advancements in the prevention and treatment of ALI, mortality rates still exceed 40% [5]. Hence, exploring the molecular mechanisms underlying the pathogenesis of ALI and identifying novel therapeutic targets for its treatment are of paramount importance.

Macrophages are key immune cells regulating pulmonary inflammation. In the early response to lung injury, lipopolysaccharide (LPS) stimulates the classical activation phenotype (AM1) macrophages to accumulate in large numbers and secrete proinflammatory cytokines, recruiting monocytes and neutrophils, thus advancing pulmonary inflammation. These pathological changes lead to epithelial–endothelial barrier damage, increased pulmonary microvascular permeability, and retention of protein-rich fluid, ultimately promoting the onset and progression of ALI [6, 7]. Conversely, selectively activated phenotype (AM2) macrophages secrete anti-inflammatory and proangiogenic factors and phagocytose apoptotic cells, transitioning toward AM1 phenotype to promote tissue repair [8–11]. Post LPS exposure, phosphorylation of dynamin-related protein 1 (DRP1) at the Ser616 site promotes mitochondrial fission, further augmenting Mito-ROS, thereby upregulating NLRP3 protein levels [12, 13]. Activation of NLRP3 can promote macrophage polarization to AM1 [14, 15]. Additionally, activated NLRP3 facilitates the activation of pro-caspase-1 and the formation of active caspase-1 p10/20 tetramers. The activated caspase-1 cleaves Gasdermin D (GSDMD), pro-IL-1*β*, and pro-IL-18 into their active forms, GSDMD-NT, IL-1*β*, and IL-18, respectively. GSDMD-NT induces membrane perforation, disrupting electrolyte homeostasis and releasing its contents, thereby triggering pyroptosis. Concurrently, the release of IL-1*β* and IL-18 exacerbates the local inflammatory response [2, 16, 17]. Therefore, NLRP3 may serve as a common mediator for macrophage polarization and pyroptosis, playing a significant role in ALI.

Mdivi-1 is a cell-permeable quinazolinone that acts as a selective inhibitor of the mitochondrial fission protein DRP1 [18]. By inhibiting DRP1-dependent mitochondrial fission, it promotes the shift of macrophages/microglia from the proinflammatory M1 phenotype to the anti-inflammatory M2 phenotype, thereby reducing the inflammatory response in experimental autoimmune encephalomyelitis (EAE) mice [19]. On the other hand, it inhibits M1 polarization mediated by Mito-ROS/NLRP3, thereby reducing the formation of atherosclerosis [15]. Additionally, DRP1 activation induces mitochondrial fission and activates the NLRP3 inflammasome-mediated pyroptosis of cardiomyocytes through a caspase-1-dependent pathway, while DRP1 knockout can improve mitochondrial function, reduce NLRP3 protein levels, and decrease cell pyroptosis [20, 21]. However, the role of Mdivi-1 in the polarization and pyroptosis of M1 alveolar macrophages in ALI remains unclear. We hypothesize that inhibiting DRP1 can reduce ALI-induced mitochondrial fission, inhibit the production of mitochondrial ROS, and thereby reduce the activation of NLRP3.

## 2. Methods and Materials

### 2.1. Animals

All animal procedures in this study were conducted in accordance with the international guidelines for the care and use of laboratory animals and were approved by the Ethics Committee of Wuhan University People's Hospital (permission number 20240505B). Male C57BL/6 mice (20–22 g) were obtained from the Hubei Provincial Research Center of Experimental Animals. All animals were housed in cages with a humidity level of 30%–37% and a temperature of 23–25°C, under a 12-h light/dark cycle. They had free access to food and water and were acclimated to the environment for 7 days before the experiments. A total of 24 wild-type C57BL/6 mice were randomly divided into three groups, including a control group, an LPS group, and an LPS + Mdivi-1 group. All drugs were administered via intraperitoneal injection as follows: Control Group: Intraperitoneal injection of an equivalent volume of physiological saline, LPS Group: Intraperitoneal injection of LPS (10 mg/kg; Sigma–Aldrich, St. Louis, MO, USA) dissolved in 0.9% physiological saline, and LPS + Mdivi-1 Group: Intraperitoneal injection of Mdivi-1 (10 mg/kg; Houston, TX, USA) 2 h after the administration of LPS (10 mg/kg). The experiment was terminated 24 h after LPS injection, and mice were anesthetized with pentobarbital sodium. Blood samples were collected from the orbital sinus, and bronchoalveolar lavage fluid (BALF) was collected. The left lower lung lobe was fixed in 4% formaldehyde for morphological evaluation. Other lung lobes were frozen at -80°C for protein blotting and biochemical analysis.

### 2.2. Wet/Dry Ratio (W/D)

The lung wet/dry weight (W/D) ratio is used to assess lung edema. After anesthetizing the mice, fresh lung tissues were collected, blotted dry and immediately weighed to obtain the wet weight (W). Subsequently, the samples were wrapped in weighing paper and placed in an oven at 60°C until a constant weight was achieved, resulting in the dry weight (D). The W/D ratio was calculated using the following formula:

  $$\begin{array}{c} W/D\times 100\%. \end{array}$$

### 2.3. Hematoxylin and Eosin (H&E) Staining

The left lower lobe of the lung was fixed in 4% formaldehyde for 24 h and subsequently embedded in paraffin. Tissue blocks were sectioned into 5 μm-thick slices, followed by staining with H&E. Tissue histopathological changes in different lung structures were observed under an optical microscope.

### 2.4. Bronchoalveolar Lavage Fluid Analysis

Following intraperitoneal injection of pentobarbital sodium anesthesia and cervical dislocation for euthanasia, the mice were subjected to tracheal intubation. The tracheal tube was inserted, and the lungs were lavaged three times with a total volume of 1 ml of PBS. The recovery rate of BALF was maintained at above 80%. After centrifuging the lavage fluid at 3000 rpm for 10 min at 4°C, the supernatant was stored in a −20°C freezer. Subsequently, levels of inflammatory cytokines (TNF-*α*, IL-1*β*, and IL-18) in the BALF were determined using enzyme-linked immunosorbent assays (ELISA), (Jianglai, China). Total protein concentration in the collected supernatant was quantified using a BCA protein assay kit (Proteintech, Wuhan, China).

### 2.5. Cell Culture

RAW264.7 alveolar macrophages were obtained from the American Type Culture Collection (ATCC, USA) and cultured in Dulbecco's modified Eagle medium (DMEM, Gibco, USA) containing 10% fetal bovine serum (FBS, Gibco, USA) and 1% penicillin. For experiments involving drug inhibitors, cells were treated with inhibitors dissolved in DMSO, using an equal volume of DMSO as a vehicle control. Cells were pretreated with 10 μM NLRP3 inhibitor (Selleck, USA), 100 μM Mito-TEMPO (Selleck, USA), or 10 μM Mdivi-1 (Selleck, USA) for 2 h, followed by stimulation with LPS (1 μg/ml) for 24 h. In the control group, RAW264.7 alveolar macrophages were treated with the vehicle. In the LPS group, RAW264.7 alveolar macrophages were treated with 1 μg/ml LPS in addition to the vehicle. For DRP1 inhibition, RAW264.7 alveolar macrophages were transfected with Si-NC control and Si-DRP1 (Guangzhou Ruibo Biotech, Guangzhou, China) at a concentration of 50 nM using Lipofectamine 3000 for 72 h. Subsequently, RAW264.7 alveolar macrophages were collected after various treatments for further experiments.

### 2.6. Flow Cytometry

Flow cytometry analysis was conducted using a flow cytometer (BD Biosciences, Franklin Lakes, NJ, USA) to study cell apoptosis via the Annexin V/PI double-staining method (Invitrogen). Transfected RAW264.7 alveolar macrophages were harvested and mixed with FITC-Annexin V and PI in 1 × binding buffer in the dark for 15 min. Apoptotic cells were detected using flow cytometry.

### 2.7. Immunofluorescence Staining

Cells subjected to various treatments were placed on glass slides for 24 h, followed by washing with PBS. Subsequently, cells were fixed with 4% paraformaldehyde (PFA) for 10 min and blocked with 5% bovine serum albumin (BSA) for 10 min. Successively, primary and secondary antibodies against F4/80, iNOS, and Arg-1 were incubated with the cells. After washing with PBS, the glass slides were stained with DAPI, and fluorescence detection was performed to observe the polarization status of the cells. Additionally, dual staining for cleaved caspase-1 immunofluorescence and terminal deoxynucleotidyl transferase dUTP nick-end labeling (TUNEL) was performed to detect cell pyroptosis. Blue fluorescence (DAPI) indicated cell nuclei, while green and red fluorescence represented TUNEL-positive cells and caspase-1, respectively. The pyroptosis rate was calculated as the ratio of TUNEL-positive and cleaved caspase-1-positive co-localized cells to DAPI-stained cells.

### 2.8. Immunohistochemistry

Paraffin-embedded tissue sections were subjected to antigen retrieval by heating in a water bath and treated with 3% hydrogen peroxide (China National Pharmaceutical Group, Beijing, China) at room temperature for 15 min to block endogenous peroxidase activity. After blocking with goat serum for 15 min at room temperature, the sections were incubated with the primary antibody against F4/80 (diluted 1:50 in PBS; Santa Cruz Biotechnology, California, USA) overnight at 4°C. Subsequently, the sections were stained with a secondary antibody, goat anti-mouse IgG labeled with horseradish peroxidase (HRP) (diluted 1:500 in PBS; ThermoFisher, Waltham, MA, USA), at 37°C for 1 h, followed by counterstaining with hematoxylin (Solarbio). Immunohistochemical staining was observed under an optical microscope (Olympus Corporation) at the original magnification of ×400.

### 2.9. Quantitative Real-Time Polymerase Chain Reaction (RT-qPCR)

Total RNA was extracted from lung tissues and macrophages using TRIzol reagent (TaKaRa, Japan). cDNA was synthesized from the harvested RNA using the PrimeScript RT Kit (Accurate Biotechnology, China) through reverse transcription, and mRNA expression levels were determined. SYBR Green Premix Pro Taq qPCR Kit (ABclonal, China) was used for PCR with specific primers for iNOS, CD86, TNF-*α*, CD206, IL-10, and *β*-actin on the CFX96 Real-Time PCR Detection System (Bio-Rad, China). Gene expression differences between different groups were analyzed using the 2^(−*ΔΔ*Ct)^ method. *β*-actin was used as the reference gene, and three independent experiments of qRT-PCR were conducted.

### 2.10. Western Blot

Total protein was extracted from fresh lung tissue and RAW264.7 cells by homogenization using RIPA lysis buffer, and protein concentration was quantified using the BCA method. The collected total proteins were separated on a 10% sodium dodecyl sulfate-polyacrylamide gel electrophoresis (SDS-PAGE) and then transferred onto a PVDF membrane. After blocking with 5% skim milk for 1 h, the PVDF membrane was incubated overnight at 4°C with one of the following primary antibodies: rabbit anti-DRP1 (CST, Cat No: 5391, 1:1000 dilution); rabbit anti-DRP1 (Phospho-Ser616) (Abcam, Cat No: ab314755, 1:1000 dilution); rabbit anti-NLRP3 (CST, Cat No: 15101S, 1:1000 dilution); rabbit anti-pro-Caspase-1 (Abcam, Cat No: ab179519, 1:1000 dilution); rabbit anti-GSDMD-NT (Cohesion, Cat No: CQA6563, 1:750 dilution); rabbit anti-GAPDH (Proteintech, 10494-1-AP, 1:10,000 dilution). The membrane was then washed with phosphate-buffered saline containing 0.1% Tween-20 for 10 min, repeated three times. Subsequently, it was incubated with a secondary IgG antibody (1:10,000) coupled with horseradish peroxidase at room temperature for 1 h. Antibody-specific protein detection was performed using an enhanced chemiluminescence detection system. Data were presented as the relative ratio of the target protein to the reference protein. The relative value of the target protein in the control group was set as 1.

### 2.11. Detection of Mito-ROS

The intracellular Mito-ROS level was detected by Mito-SOX Red Mitochondrial Superoxide Indicator (Beyotime, China). After incubation with diluted Mito-SOX for 5 min at room temperature, macrophages were washed by 1× PBS 3 times. The red fluorescence intensity is positively correlated with the production of ROS in mitochondria and is observed and photographed using a laser confocal microscope.

### 2.12. Statistical Analysis

Each experiment was conducted in triplicate. All data are presented as the mean ± standard deviation. Student's *t*-test was used for comparisons between two groups. One-way analysis of variance (ANOVA) was employed to analyze differences among multiple groups. Statistical analysis was performed using SPSS 19.0 software (IBM, Armonk, NY, USA). A value of *p*  < 0.05 was considered statistically significant.

## 3. Results

### 3.1. Mdivi-1 Alleviates Pulmonary Pathological Changes and Inflammatory Response in Sepsis-Induced ALI Mice

**Figure 1:**
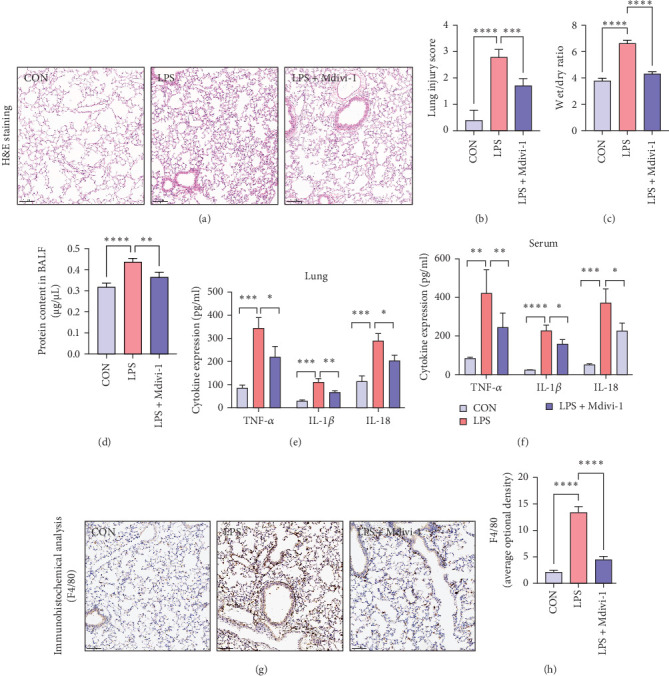
Mdivi-1 alleviates lung pathological changes and inflammatory response in sepsis-induced ALI. (A) Histopathology of lung tissues in mice after intraperitoneal LPS injection with or without treatment with Mdivi-1 (DRP1 inhibitor). (B) Lung injury scores determined based on the severity of lung damage. Lung edema assessed by (C) wet-to-dry (W/D) ratio and (D) bronchoalveolar lavage fluid (BALF) protein concentration. (E,F) ELISA was used to detect the expression levels of inflammatory cytokines IL-1*β*, IL-18, and TNF-*α* in lung tissue homogenates and serum. (G) Representative immunohistochemical staining images of lung sections for DAPI (blue) and F4/80 (brown). Bars = 50 μm. (H) Semi-quantitative analysis of F4/80 immunohisto-chemistry. Data were shown as means ± SD. *n* = 6. *⁣*^*∗*^*p* < 0.05, *⁣*^*∗∗*^*p* < 0.01, *⁣*^*∗∗∗*^*p* < 0.001, *⁣*^*∗∗∗∗*^*p* < 0.0001.

To investigate the role of Mdivi-1 in ALI, pulmonary histopathological changes were assessed using H&E staining. Compared to the control group, mice in the LPS group exhibited severe inflammation cell infiltration and alveolar structural damage in the lungs (Figure 1A) (*p* < 0.05). Treatment with Mdivi-1 significantly ameliorated these histological alterations. The lung injury score confirmed the statistically significant therapeutic effect of Mdivi-1 (Figure 1B) (*p* < 0.05). Furthermore, lung edema was evaluated by measuring the lung W/D weight ratio and BALF protein concentration. As shown in Figure 1C,D, the lung W/D ratio and BALF protein concentration were significantly increased in the LPS group, while Mdivi-1 treatment reduced these parameters (*p* < 0.05). ELISA analysis revealed a significant increase in proinflammatory cytokines (TNF-*α*, IL-1*β*, and IL-18) in lung tissue homogenates and serum of the LPS group, which was attenuated by Mdivi-1 treatment (Figure 1E, F) (All *p* < 0.05). Immunohistochemistry results showed a significant increase in F4/80 (macrophage marker) content in the LPS group, which was markedly reduced after Mdivi-1 treatment (Figure 1G,H) (All *p* < 0.05).

### 3.2. Mdivi-1 Mitigates ALI by Suppressing M1 Macrophage Polarization

**Figure 2:**
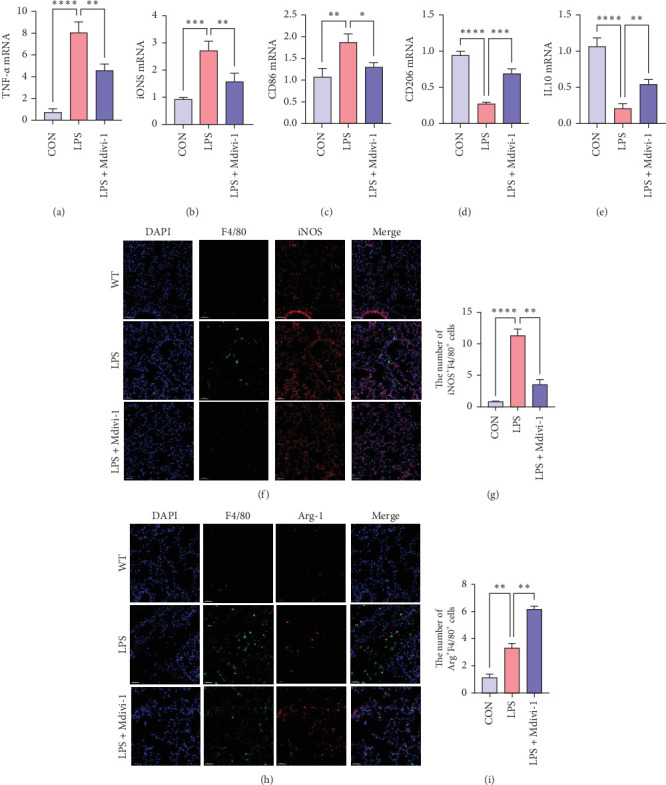
Mdivi-1 inhibits M1 polarization in sepsis-induced ALI. (A–E) qRT-PCR was used to detect mRNA expression of M1 polarization markers, including TNF-*α*, iNOS, and CD86, and M2 polarization markers, including CD206 and IL-10. Immunofluorescence used to detect (F,G) iNOS and F4/80 and (H,I) Arg-1 and F4/80 expression and colocalization after ALI. Bars = 20 μm. Data were shown as means ± SD. *n* = 3. *⁣*^*∗*^*p* < 0.05, *⁣*^*∗∗*^*p* < 0.01, *⁣*^*∗∗∗*^*p* < 0.001, *⁣*^*∗∗∗∗*^*p* < 0.0001.

To elucidate the role of Mdivi-1 in modulating pulmonary alveolar macrophage polarization and pyroptosis associated with sepsis-induced ALI, qRT-PCR was used to assess the mRNA levels of TNF-*α*, iNOS, CD86 (M1 macrophage markers), CD206, and IL-10 (M2 macrophage markers) following Mdivi-1 treatment. As shown in Figure 2A–E, compared to the control group, the mRNA levels of CD86, iNOS, and TNF-*α* in the lungs of LPS-induced mice were significantly increased. Mdivi-1 treatment downregulated the mRNA levels of CD86, iNOS, and TNF-*α*, while it increased CD206 and Arg-1 levels (All *p* < 0.05). Immunofluorescence confirmed the enhanced protein expression and co-localization of iNOS and F4/80 (Figure 2F,G) in ALI, while the expression and co-localization of Arg-1 and F4/80 (Figure 2H,I) (All *p* < 0.05) were significantly reduced. These adverse effects were markedly reversed by Mdivi-1 treatment.

### 3.3. Mdivi-1 Mitigates ALI by Suppressing Macrophage Pyroptosis

**Figure 3:**
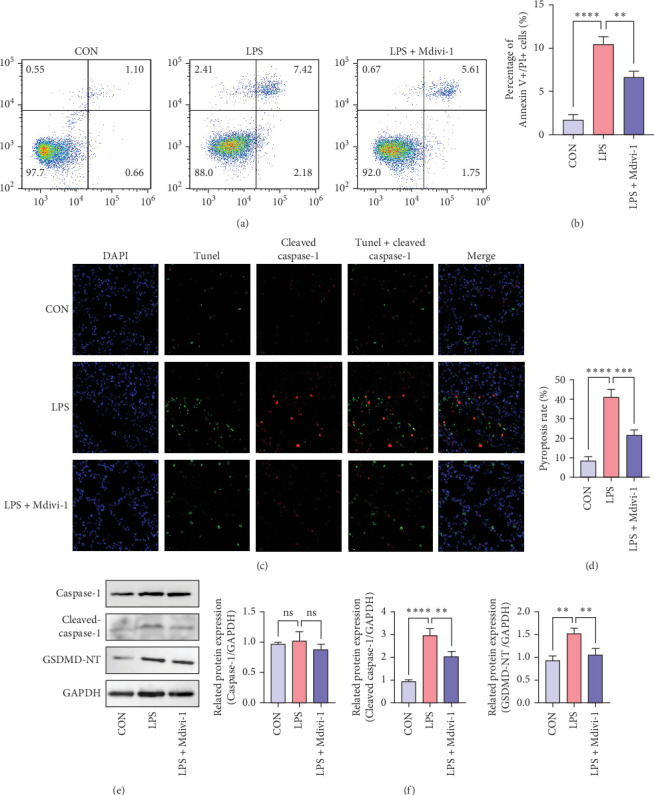
Mdivi-1 inhibits pyroptosis of alveolar macrophages in sepsis-induced ALI. (A, B) Representative flow cytometry plots of RAW264.7 membrane-associated protein V/PI staining and analysis of double-stained cells for membrane-associated protein V/PI. (C, D) Representative immunofluorescence images of pyroptosis in RAW264.7 cells (caspase-1/TUNEL double-positive cells) and analysis of pyroptosis rate in RAW264.7 cells. Bars = 20 μm. (E) Western blot showing protein levels of pyroptosis-related proteins (caspase-1, cleaved caspase-1 and GSDM-T). (F) Relative protein changes for caspase-1/GAPDH, cleaved caspase-1/GAPDH and GSDMD-NT/GAPDH. Data were shown as means ± SD. *n* = 3. *⁣*^*∗∗*^*p* < 0.01, *⁣*^*∗∗∗*^*p* < 0.001, *⁣*^*∗∗∗∗*^*p* < 0.0001, and ns *p*  > 0.05.

We found that flow cytometry analysis revealed a significant increase in apoptotic cells (Annexin V + PI+) in Raw264.7 alveolar macrophages following LPS treatment (Figure 3A,B) (All *p* < 0.05). To further characterize the type of cell death in RAW267.7 cells, immunofluorescence was used to detect the co-expression of Tunel and cleaved caspase-1 (a characteristic of pyroptosis) in alveolar macrophages. The results showed that cell pyroptosis increased after 24 h of intraperitoneal LPS injection, and in the Mdivi-1 treatment group, cell pyroptosis was reduced (Figure 3C,D) (All *p* < 0.05). Through Western blot analysis, it was found that LPS significantly stimulated an increase in the expression of pyroptosis related proteins (Cleaved caspase-1 and GSDMD-NT). After Mdivi-1 treatment, the expression levels of these pyroptosis proteins correspondingly decreased (Figure 3E,F) (All *p* < 0.05). The full membrane images of Western Blot can be found in Figure S1. All these data suggest that Mdivi-1 may inhibit M1 macrophage activation and pyroptosis in vivo, thereby alleviating lung inflammation.

### 3.4. Mdivi-1 Suppresses Phosphorylation of DRP1 (Ser616) to Alleviate ALI in Sepsis by Inhibiting NLRP3 Activation

**Figure 4:**
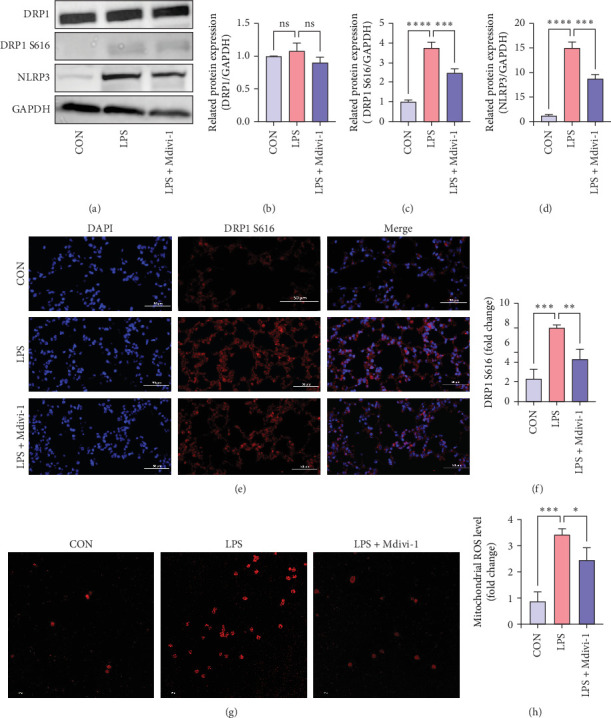
DRP1 promotes phosphorylation of DRP1 (Ser616), activation of NLRP3, and mitochondrial ROS increase in lung tissues of septic mice. (A) Western blot showing protein levels of total DRP1, phosphorylated DRP1 (Ser616) and NLRP3. (B–D) Relative protein changes for DRP1/GAPDH, phosphorylated DRP1 (Ser616)/GAPDH and NLRP3/GAPDH. (E) Representative immunofluorescence images of phosphorylated DRP1 S616. Bars = 50 μm. (F) Analysis of phosphorylated DRP1 S616 in lung tissue. (G,H) Mito-SOX was used to detect mitochondrial ROS production. Bars = 100 μm. Data were shown as means ± SD. *n* = 3. *⁣*^*∗*^*p* < 0.05, *⁣*^*∗∗*^*p* < 0.01, *⁣*^*∗∗∗*^*p* < 0.001, *⁣*^*∗∗∗∗*^*p* < 0.0001, and ns *p* > 0.05.

To investigate its effects and potential mechanisms, the protein levels of phosphorylated DRP1 (Ser616), DRP1, and NLRP3 were examined. In the ALI group, levels of phosphorylated DRP1 (Ser616) and NLRP3 were elevated compared to the control group but were significantly downregulated after Mdivi-1 treatment (Figure 4A,B) (All *p* < 0.05). The full membrane images of Western Blot can be found in Figure S2. After LPS treatment, the immunofluorescence of phosphorylated DRP1 (Ser616) increased, and Mdivi-1 treatment can inhibit the expression of phosphorylated DRP1 (Ser616) (Figure 4C,D) (All *p* < 0.05). Additionally, the expression of mitochondrial ROS is regulated similarly and after Mdivi-1 treatment, the expression level correspondingly decreased (Figure 4E,F) (All *p* < 0.05). These results suggest that Mdivi-1 can alleviate ALI by inhibiting NLRP3 inflammasome activation and abnormal accumulation of mitochondrial ROS. However, the specific mechanism still needs to be explored in vitro.

### 3.5. LPS Induces M1 Polarization, NLRP3 Activation, and Mitochondrial ROS Accumulation in RAW264.7 Cells

**Figure 5:**
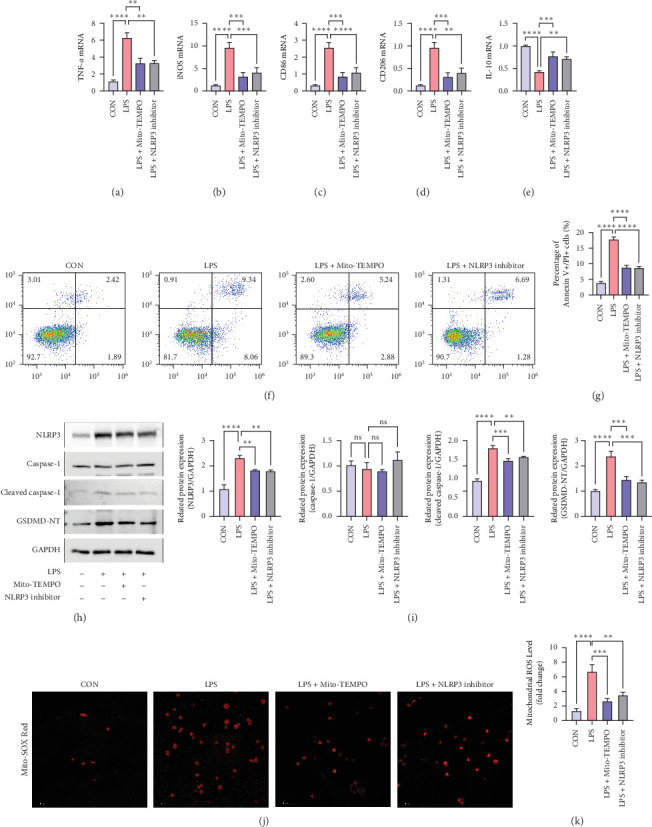
LPS promotes activation and pyroptosis of M1 alveolar macrophages through the Mito-ROS/NLRP3 pathway *in vitro*. Pre-treatment of alveolar macrophages with 10 μM NLRP3 inhibitor and 100 μM Mito-TEMPO (mitochondrial ROS scavenger) for 2 h, followed by LPS (1 μg/ml) stimulation of RAW264.7 alveolar macrophages for 24 h, with an equal volume of PBS as the negative control. (A–E) qRT-PCR detection of mRNA expression of M1 polarization markers, including TNF-*α*, iNOS and CD86, and M2 polarization markers, including CD206 and IL-10. (F,G) Flow cytometry assessment of cell death by Annexin-V and PI double staining and analysis of double-stained cells for membrane—associated protein V/PI. (H,I) Western blot analysis of NLRP3, caspase-1, cleaved caspase-1, and GSDMD-NT protein expression. (J,K) Mito-SOX detection of mitochondrial ROS levels in RAW264.7 alveolar macrophages. Bars = 100 μm. Data were shown as means ± SD. *n* = 3. *⁣*^*∗∗*^*p* < 0.01, *⁣*^*∗∗∗*^*p* < 0.001, *⁣*^*∗∗∗∗*^*p* < 0.0001 and ns *p* > 0.05.

To confirm the effects of LPS on macrophage polarization and pyroptosis, as well as its potential mechanisms, RAW264.7 cells were first pretreated with Mito-TEMPO and NLRP3 inhibitor for 2 h, followed by LPS treatment for 24 h. The mRNA expression of M1 markers (TNF-*α*, iNOS, and CD86) was significantly higher than in the control group. Furthermore, in LPS-induced RAW264.7 cells, the expression of M2 markers (CD206 and IL- 10) was suppressed (Figure 5A–E) (All *p* < 0.05). Additionally, we found that flow cytometry analysis revealed a significant increase in apoptotic cells (Annexin V + PI+) in Raw264.7 alveolar macrophages following LPS treatment, after treatment with the appeal inhibitor, the apoptosis rate of cells decreased. (Figure 5F,G) (All *p* < 0.05). Moreover, compared to the control group, LPS treatment increased the protein expression of NLRP3, cleaved-caspase-1, and GSDMD-NT (Figure 5H) (All *p* < 0.05) and led to elevated mitochondrial ROS levels (Figure 5I,J) (All *p* < 0.05). The full membrane images of Western Blot can be found in Figure S3. Both Mito-TEMPO and NLRP3 inhibitor suppressed the activation of NLRP3 inflammasome by downregulating abnormal accumulation of mitochondrial ROS, the protein levels of NLRP3, and cleaved-caspase-1 in LPS-treated RAW264.7 cells. These results indicate that the Mito-ROS/NLRP3 pathway mediates M1 macrophage polarization and pyroptosis and contributes to ALI.

### 3.6. DRP1 Knockdown Reduces M1 Polarization and Pyroptosis-Mediated ALI by Inhibiting the Mito-ROS/NLRP3 Pathway

**Figure 6:**
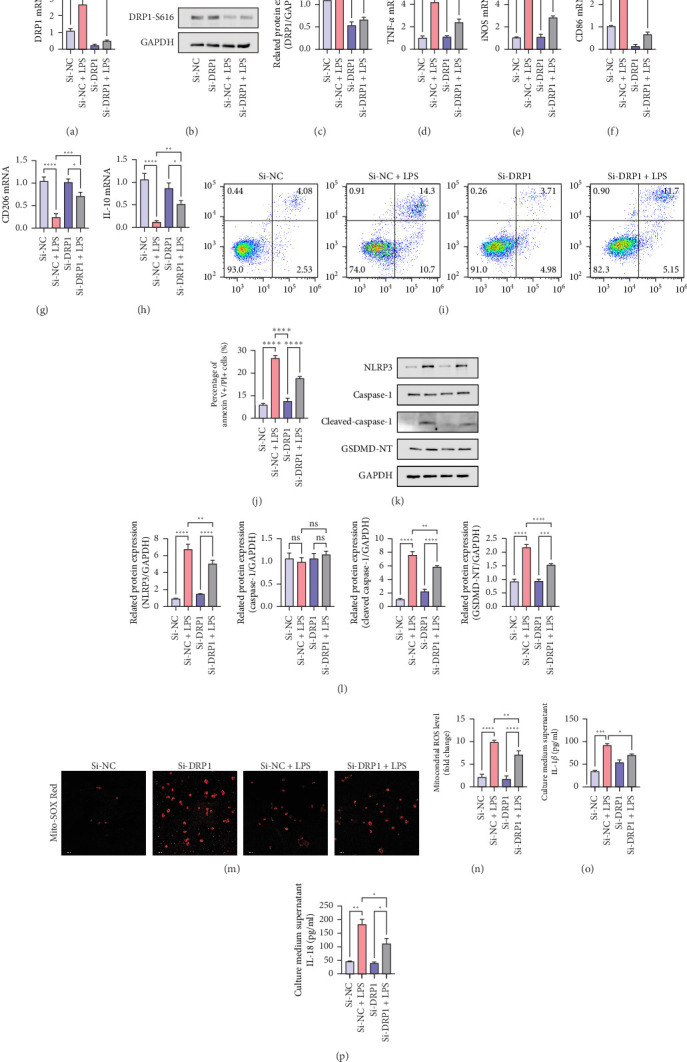
Mdivi-1 promotes M1 polarization and pyroptosis through the Mito—ROS/NLRP3 pathway. RAW264.7 macrophages were transfected with Si-NC (negative control) or Si-DRP1 for 72 h before co-culture with LPS for 24 h. (A) RT-qPCR detection of DRP1 mRNA expression. (B,C) Western blot analysis of total DRP1. (D–H) RT—qPCR detection of mRNA expression of inflammatory cytokines (TNF-*α*, iNOS, CD86, CD206, and IL-10). (I,J) Representative flow cytometry plots of membrane-associated protein V/PI staining in Si-DRP1 cells and analysis of double-stained cells for membrane-associated protein V/PI. (K,L) Western blot analysis of NLRP3, caspase-1, cleaved caspase-1, and GSDMD-NT expression. (M,N) Mito-SOX detection of mitochondrial ROS levels. RAW 264.7 macrophages were transfected with Si-NC (negative control) or Si-DRP1 for 72 h, followed by stimulation with LPS for 12 h and collection culture medium supernatant. (O,P) ELISA to measure IL- 1*β* and IL-18 levels in cell culture supernatants. Data were shown as means ± SD. *n* = 3. *⁣*^*∗*^*p* < 0.05, *⁣*^*∗∗*^*p* < 0.01, *⁣*^*∗∗∗*^*p* < 0.001, *⁣*^*∗∗∗∗*^*p* < 0.0001.

We investigated the effect of DRP1 knockdown on M1 polarization, pyroptosis, and its potential mechanisms. Si-DRP1 reduced the mRNA levels of DRP1 (Figure 6A) (*p* < 0.05) and the protein expression of DRP 1 (Figure 6B,C) (All *p* < 0.05). The full membrane images of Western Blot can be found in Figure S4. In LPS-stimulated macrophages, DRP 1 knockdown downregulated the mRNA levels of M1 markers (TNF-*α*, iNOS, and CD86) but upregulated the mRNA levels of M2 markers (CD206 and IL-10) (Figure 6D–H) (All *p* < 0.05). Additionally, we found that flow cytometry analysis revealed a significant increase in apoptotic cells (Annexin V + PI+) in Raw264.7 alveolar macrophages following LPS treatment (Figure 6I,J) (All *p* < 0.05). Furthermore, DRP1 knockdown inhibited the expression of NLRP3, cleaved caspase-1 and GSDMD-NT (Figure 6K–O) (All *p* < 0.05). The full membrane images of Western Blot can be found in Figure S5. Similarly, in vitro culture with Si-DRP 1 knockdown, it was found that Mito ROS expression levels were reduced (Figure 6P,Q) (All *p* < 0.05). After 72 h of transfection with Si-DRP1, the cells were stimulated with LPS for 24 h. Cell culture medium was collected, and ELISA was used to detect the levels of IL-1 *β* and IL-18 relative decrease (Figure 6R,S) (All *p* < 0.05). These results indicate that downregulation of DRP1 expression can alleviate M1 polarization and pyroptosis-mediated ALI by inhibiting the Mito-ROS/NLRP3 pathway.

## 4. Discussion

This study investigates the protective effects of the DRP1 inhibitor Mdivi-1 on sepsis-induced ALI in mice and explores its potential mechanisms in modulating macrophage polarization and alleviating macrophage pyroptosis. An ALI mouse model was established through intraperitoneal injection of LPS. These mice exhibited typical pathological characteristics of ALI, such as pulmonary edema, histopathological alterations in lung tissues, and infiltration of inflammatory cells. The mice in the LPS + Mdivi-1 group displayed significantly lower lung injury scores, levels of inflammatory cytokines, and W/D ratios compared to the LPS group. Mdivi-1 treatment mitigated the extent of inflammatory damage and preserved organ function, substantiating its protective role in LPS-induced ALI. Notably, the anti-ALI action of Mdivi-1 in reducing M1 alveolar macrophage polarization and pyroptosis may be associated with the inhibition of the Mito-ROS/NLRP3 pathway.

Alveolar macrophages, accounting for 90% of the cells in BALF, serve as the first line of defense against pathogen invasion during sepsis [22, 23]. Upon bacterial and LPS stimulation, resting macrophages undergo polarization, releasing a plethora of cytokines such as tumor necrosis factor-alpha (TNF-*α*), IL-1*β*, IL-6, and ROS, which are pivotal pathogenic factors in ALI [24]. Presently, the modulation of macrophage polarization is an emerging target for ALI treatment. LPS can induce M1 polarization in alveolar macrophages, upregulating M1 markers (CD86, iNOS, and TNF-*α*) and downregulating M2 markers (CD206 and IL-10), thereby exacerbating pulmonary inflammation [25]. This study demonstrates that Mdivi-1 intervention attenuates the in vivo LPS-driven inflammatory response by inhibiting M1 alveolar macrophage polarization. In vitro treatment with Mdivi-1 and downregulation of DRP1 can inhibit ALI by suppressing the activation of RAW264.7 alveolar macrophages stimulated by LPS.

Pyroptosis is primarily a caspase-1-dependent cell death. Activated caspase-1 cleaves GSDMD to produce GSDMD-NT, inducing pore formation in membranes, leading to cell swelling, plasma membrane rupture, and release of proinflammatory cellular contents [26]. Our findings reveal a significant upregulation of LPS-induced macrophage pyroptosis proteins cleaved caspase-1 and GSDMD-NT upon nigericin treatment. Similarly, the levels of IL-1*β* and IL-18 in the culture medium of nigericin and LPS-stimulated macrophages were significantly elevated. However, these changes were markedly reversed by downregulating DRP1 levels.

The activation of the NLRP3 inflammasome and the generation of proinflammatory cytokines IL-1*β* and IL-18 in alveolar macrophages play a crucial role in alveolar macrophage pyroptosis during ALI [27, 28]. Moreover, the NLRP3 inflammasome also exerts a crucial role in various inflammatory diseases by modulating M1 polarization [14, 29–31]. However, whether inhibiting the activation of the NLRP3 inflammasome can suppress ALI by modulating M1 alveolar macrophage polarization and pyroptosis remains unclear.

In this study, M1 alveolar macrophage polarization, pyroptosis, and NLRP3 activation were observed in the lung tissues of LPS mice. Similar findings were observed in vitro, indicating that M1 alveolar macrophage polarization, pyroptosis, and NLPR3 activation are involved in the pathogenesis and progression of ALI. Moreover, NLRP3 inhibitors significantly suppressed LPS-induced M1 alveolar macrophage polarization and cell pyroptosis. In summary, our results suggest that NLRP3-dependent M1 alveolar macrophage polarization, and cell pyroptosis may play a significant role in ALI. Previous studies have indicated that the generation of Mito-ROS is a critical mechanism for NLRP3 activation [31, 32]. Post LPS stimulation, DRP1 binds with fission 1 and mitochondrial fission factor to mediate metabolic disorder, inhibit glutathione in mitochondria, weaken the capacity to clear free radicals, further increase Mito-ROS, thereby upregulating NLRP3 protein levels and causing NLRP3 inflammasome activation [33]. In this study, we found that the mito-ROS scavenger Mito-TEMPO could reduce M1 alveolar macrophage polarization and cell pyroptosis-mediated inflammatory responses by inhibiting NLRP3 activation, suggesting that mito-ROS/NLRP3-dependent M1 alveolar macrophage polarization and cell pyroptosis may promote the onset and progression of ALI.

During ALI, endotoxin leads to a 31% increase in systemic ROS, accompanied by excessive mitochondrial fission and damage to alveolar macrophages [34]. DRP1, a core molecule controlling mitochondrial fission, stimulates DRP1 translocation from the cytosol to mitochondria and promotes fission when phosphorylated at serine 616. Conversely, phosphorylation at serine 637 inhibits fission [35]. Studies have shown that Mdivi-1 can alleviate LPS-induced ALI by inhibiting the phosphorylation of DRP 1 at the Ser616 site, hindering DRP1 translocation from the cytosol to mitochondria, and promoting phosphorylation at the Ser637 site [36]. Additionally, Mdivi-1 also inhibits mitochondrial autophagy and protects rats from LPS-induced cell apoptosis, oxidative stress, and inflammation in ALI [37]. However, the potential relationship between Mdivi-1, macrophage polarization, and pyroptosis in ALI has not been reported. Our study indicates that Mdivi-1 inhibits the expression of DRP1 Ser616, subsequently suppressing NLRP3 activation and M1 alveolar macrophage polarization in the lung tissues of ALI mice. Furthermore, inhibiting DRP1-dependent fission through Mdivi-1 or DRP1 knockdown significantly reduces the abnormal accumulation of Mito-ROS and inhibits NLRP3 activation, thereby alleviating M1 alveolar macrophage polarization and pyroptosis-mediated ALI. In summary, our findings suggest that Mdivi-1 can reduce M1 alveolar macrophage polarization and pyroptosis-mediated ALI by modulating the Mito-ROS/NLRP3 inflammasome signaling pathway.

Alveolar macrophage polarization and pyroptosis are key processes in the onset and progression of ALI. Previous research has shown that in LPS-induced ALI mice, the polarization of M1 alveolar macrophages increases, and the expression of cell pyroptosis proteins (such as cleaved caspase-1 and GSDMD) in lung tissues is elevated. Further research discovered that rTsP53 protein in mice can promote M2 macrophage polarization, reducing cell pyroptosis in lung tissues, thereby protecting against lung injury [38]. Additionally, studies have found that anti-inflammatory M2 macrophages can reduce inflammation-induced cell pyroptosis, thereby alleviating LPS-induced lung injury [6, 39]. Therefore, we speculate that M1 and M2 alveolar macrophages may participate in the pathogenesis and progression of ALI by influencing the release of inflammatory factors and cell pyroptosis. However, the specific mechanisms of alveolar macrophage polarization and cell pyroptosis are not yet clear, and whether Mdivi-1 modulates cell pyroptosis by regulating the polarization of M1 alveolar macrophages warrants further investigation.

## 5. Conclusion

Our research indicates that DRP1 activation in ALI is tied to the Mito ROS/NLRP3 inflammasome pathway, which is crucial in inflammation. Increased Mito ROS can trigger NLRP3 inflammasome activation, leading to the release of inflammatory cytokines IL-1*β* and IL-18. This process may enhance M1 macrophage polarization in sepsis-induced lung inflammation, promoting a proinflammatory environment and macrophage pyroptosis, which can exacerbate lung damage. Mdivi-1′s potential to reduce inflammation by inhibiting DRP1-dependent mitochondrial fission suggests it could be a therapeutic option. Further study is needed to explore Mdivi-1′s mechanism and effectiveness in treating sepsis-related ALI, offering new therapeutic prospects by targeting these pathways.

## Data Availability

All data supporting the findings of this study are available within the paper and its Supporting Information. Raw data of western blot whole membraneare provided in Supporting Information S1–S5. If additional raw data are needed, it can be obtained with the consent of the corresponding author.

## Nomenclature

DRP1: Dynamin-related protein 1
Mito-ROS: Mitochondrial reactive oxygen species
ALI: Acute lung injury
ARDS: Acute respiratory distress syndrome
mtROS: Mitochondrial reactive oxygen species
LPS: Lipopolysaccharide
NLRP3: NOD-like receptor pyrin domain containing 3
BALF: Bronchoalveolar lavage fluid.

## Supporting Information

Supporting Information The supporting information Figures S1–S5 include full Western Blot membrane images, showing antibody detection results for all experiments. These images provide complete membrane scans for each sample, ensuring transparency and reproducibility. All images are labeled with molecular weight markers, target protein bands, and are arranged in the order described in the main text.
